# Exchange-bias *via* nanosegregation in novel Fe_2−*x*_Mn_1+*x*_Al (*x* = −0.25, 0, 0.25) Heusler films†

**DOI:** 10.1039/c9na00689c

**Published:** 2020-05-01

**Authors:** S. Kurdi, M. Ghidini, G. Divitini, B. Nair, A. Kursumovic, P. Tiberto, S. S. Dhesi, Z. H. Barber

**Affiliations:** Department of Materials Science and Metallurgy, University of Cambridge CB3 0FS Cambridge UK sk862@cantab.ac.uk; Department of Physics, Mathematics and Computer Science, University of Parma 43130 Parma Italy massimo.ghidini@unipr.it; Diamond Light Source Chilton Didcot OX11 0DE Oxfordshire UK; The National Institute for Metrological Research (INRIM) 10135 Torino Italy

## Abstract

Exchange-bias has been reported in bulk nanocrystalline Fe_2_MnAl, but individual thin films of this Heusler alloy have never been studied so far. Here we study the structural and magnetic properties of nanocrystalline thin films of Fe_2−*x*_Mn_1+*x*_Al (*x* = −0.25, 0 and 0.25) obtained by sputtering and *ex situ* post-deposition annealing. We find that Fe_2_MnAl films display exchange-bias, and that varying Mn concentration determines the magnitude of the effect, which can be either enhanced (in Fe_1.75_Mn_1.25_Al) or suppressed (in Fe_2.25_Mn_0.75_Al). X-ray diffraction shows that our films present a mixed L2_1_–B2 Heusler structure where increasing Mn concentration favors the partial transformation of the L2_1_ phase into the B2 phase. Scanning transmission electron microscopy (STEM) and energy dispersive X-ray spectroscopy (EDX) reveal that this composition-driven L2_1_ → B2 transformation is accompanied by phase segregation at the nanoscale. As a result, the Fe_2−*x*_Mn_1+*x*_Al films that show exchange-bias (*x* = 0, 0.25) are heterogeneous, with nanograins of an Fe-rich phase embedded in a Mn-rich matrix (a non-negative matrix factorisation algorithm was used to give an indication of the phase composition from EDX data). Our comparative analysis of XRD, magnetometry and X-ray magnetic circular dichroism (XMCD), shows that the Fe-rich nanograins and Mn-rich matrix are composed of a ferromagnetic L2_1_ phase and an antiferromagnetic B2 phase, respectively, thus revealing that exchange-coupling between these two phases is the cause of the exchange-bias effect. Our work should inspire the development of single-layer, environmentally friendly spin valve devices based on nanocomposite Heusler films.

## Abstract 2

Exchange-bias has been reported in bulk nanocrystalline Fe_2_MnAl, but individual thin films of this Heusler alloy have never been studied so far.

## Graphical abstract

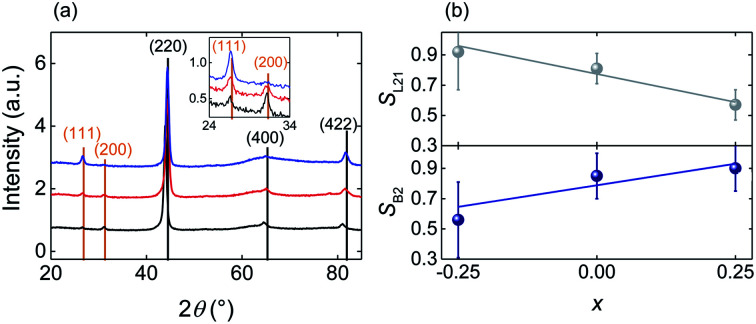

## Introduction

For a sustainable economy, the development of environmentally friendly magnetic applications is a priority.^1,2^ In this field, magnetic materials for energy applications (*i.e.* permanent magnets and magnetocalorics) have insofar been at the forefront, with focus on reducing cost by replacing rare with abundant elements,^3,4^ and on exploring simpler and greener device designs.^5^ However, the same criteria can be extended to other applications.

Magnetic field sensors based on the spin-valve device concept require a multilayer stack comprising two soft ferromagnetic (FM) layers and an antiferromagnetic (AFM) layer (usually IrMn). The magnetization of the outer FM layer is free to rotate in response to external magnetic fields, while the magnetization of the inner FM layer is pinned to the underlying AFM layer *via* the exchange-bias effect.^6^ A simpler, cost-effective device could be obtained if the multilayer stack were replaced with a single layer of material with exchange-bias. For optimum sustainability, this single layer should contain neither Ir (one of the scarcest elements in the earth's crust) nor any other scarce or hazardous material.^7^

Exchange-bias has long been known in heterogeneous granular systems that present the necessary coexistence of AFM and FM phases at the nanoscale.^8^ These systems have been shown to work at room temperature^9^ and have been proposed for spintronics applications. Suitable morphologies comprise: (i) core–shell nanoparticles obtained by mechanical milling,^10^ thermal decomposition^11^ and different chemical routes;^12–14^ (ii) magnetic nanoparticles embedded in AFM matrices obtained by cluster beam deposition,^15–17^ selective reduction^18^ and co-sputtering;^19^ and (iii) phase segregated alloys obtained by mechanical^20^ and thermal^21–23^ treatments.

Moreover, exchange-bias has been reported in alloys with a nominally homogeneous composition, where the separation between FM and AFM phases can be intrinsically established by structural phase transformations^24^ and/or chemical disorder.^25,26^ These conditions occur in Mn-based Heusler alloys that present the shape memory effect,^27–35^ compensated ferrimagnetism^36^ and spin glass behaviour.^21,37,38^ The observation of exchange-bias in nominally single Heusler alloys is intriguing for spintronic devices because of their highly spin-polarized conduction band.^39–42^

The Heusler alloy Fe_2_MnAl is promising for application in environmentally friendly spin valve devices because it is ferromagnetic at room temperature,^43,44^ it is made of abundant non-hazardous materials and can be prepared as core/shell nanoparticles that display exchange-bias.^45^ However, Fe_2_MnAl has not been studied in the form of individual thin films to date.

Here, we report on the structural and magnetic properties of nanocrystalline thin films of Fe_2−*x*_Mn_1+*x*_Al (*x* = −0.25, 0, 0.25) obtained by sputtering and *ex situ* post-deposition annealing. We show that our films display a mixed L2_1_–B2 Heusler structure in which substitution of Mn for Fe progressively drives the L2_1_ → B2 phase transformation. Macroscopic magnetic measurements reveal that this transformation favours exchange-bias, but depresses magnetization. A detailed STEM investigation shows that these crystallographic and magnetic changes are the result of a nanosegregation process, which ultimately leads to a heterogeneous microstructure in which coarsened crystal grains of an Fe-rich phase are embedded in a Mn-rich phase. We use a non-negative matrix factorisation (NMF) algorithm^46,47^ to give an indication of the composition of the two phases. Moreover, we clarify the magnetic nature of the phases *via* a quantitative comparison between macroscopic and microscopic magnetic measurements of the Fe_1.75_Mn_1.25_Al film. XMCD measurements yield an overall magnetic moment which exceeds, by a factor of two, the value obtained from the (macroscopic) saturation magnetization calculated over the whole volume of the film, measured by SQUID magnetometry. However, we find good agreement when we use the volume of the Fe-rich phase as determined from the STEM images, such that we are able to identify the Fe-rich phase as the L2_1_ ferromagnetic phase and the Mn-rich phase as the antiferromagnetic B2 phase. In this heterogeneous L2_1_–B2 mixed state, the exchange-bias results from the exchange-coupling between phase-separated ferromagnetic and antiferromagnetic regions. Our environmentally friendly nanocomposite Heusler films should be of interest for developing single-layer spin valve devices which are sustainable.

## Experimental

Fe_2−*x*_Mn_1+*x*_Al films (*x* = −0.25, 0 and 0.25) with a thickness of 200 nm and 2.5 nm-thick Al capping layers were grown by multi-target DC-magnetron sputtering on thermally oxidized Si substrates. After reaching a base pressure of 1 × 10^−7^ Pa in the deposition system, the films and capping layers were deposited at a rate of 0.06 nm s^−1^ and 0.017 nm s^−1^ respectively (in a 99.999% Ar atmosphere with a pressure of 1.37 Pa). The power supplied to each of the (elemental) magnetron targets was carefully controlled in order to control the individual deposition rates, and hence the film composition. The samples were subsequently annealed *ex situ* at 300 °C for 9 h in a vacuum of 1 × 10^−6^ Pa to induce structural ordering.

After annealing, the nominal compositions (calculated from the deposition rates of the individual elements) were confirmed by energy dispersive X-ray analysis (EDX) using a Camscan MX2600 FEG-SEM. To rule out any possible artefacts arising from the interaction of the electron beam with the substrate in these 200 nm-thick films, subsequently used for the magnetic studies, compositions were also confirmed in 600 nm-thick twin samples that were specifically grown for this purpose.

The surface morphology of the films was studied by tapping mode atomic force microscopy (AFM) (Bruker, MM8 with a Nanoscope V controller). We used TAP300AL Budget Sensors cantilevers and we analysed the images using the Gwyddion software package.^48^

**Fig. 1:**
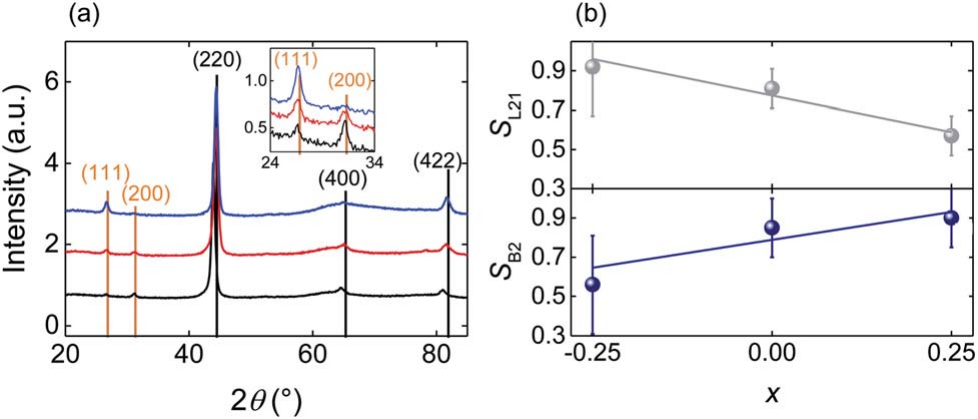
Structural characterization of the annealed Fe_2−*x*_Mn_1+*x*_Al films. (a) Full (main panel) and zoomed (inset) XRD scans for Fe_2.25_Mn_0.75_Al (blue), Fe_2_MnAl (red) and Fe_1.75_Mn_1.25_Al (black) compositions. Fundamental and superlattice reflections are labelled in black and orange respectively. (b) Order parameters^56^*S*_L2_1__ and *S*_B2_ as a function of composition *x*. Error bars represent the standard errors determined by repeating the measurements on three equivalent films for each value of *x*.

Structural characterisation was carried out by X-ray diffraction (XRD) using a Bruker D8 Advance diffractometer. To eliminate the substrate peaks we used an off-coupled *θ*/2*θ* scan with an 8° *θ* offset, which did not affect the intensity of the diffraction peaks, given that our films were untextured. The experimental peak intensities were determined by profile fitting using HighScore+ software. The theoretical intensities corresponding to the fully ordered crystals that we have used for our quantitative analysis (Fig. 1) were calculated using the VESTA^49^ software package.

The film morphology and elemental spatial distribution were analysed with a FEI Osiris scanning transmission electron microscope (STEM) operated at 200 kV. The microscope was equipped with a high brightness electron gun and a large area EDX detector (Bruker Super-X, 0.9 sr solid angle for collection). Images were acquired in STEM-HAADF (High Angle Annular Dark Field) mode, in which the brightness depends upon the local thickness and average atomic number. For STEM studies, Fe_2−*x*_Mn_1+*x*_Al (*x* = −0.25, 0, 0.25) films with a 50 nm thickness and 2.5 nm Al capping layers were deposited and annealed *ex situ* (under the same conditions as above) on commercial TEM sample supports made of 200 nm-thick SiN membranes on 200 μm-thick Si chips with a 0.1 × 0.1 mm^2^ window. The EDX data were analyzed using a NMF (Non-negative Matrix Factorisation) algorithm^32^ run inside Hyperspy,^50^ an open-source python-based toolkit for the analysis of electron microscopy data. The NMF algorithm improves the signal-to-noise ratio (SNR) and enables correlations between the spatial distribution of elements to be identified. Such correlations emerge from statistical analysis and consist of maps that describe the distribution of chemical compounds rather than just elements. The algorithm can be thought of as a generalised fitting routine in which a set of EDX spectra are modelled into a linear combination of different factors (which can be both single elements and chemical compounds), weighted by a set of corresponding loadings. The loading for each pixel represents the weight in the linear combination that reconstructs the original dataset and thus indicates the presence of a given component (and therefore the associated compound) in that pixel.

All the magnetic measurements were carried out on the 200 nm-thick Fe_2−*x*_Mn_1+*x*_Al films.

Hysteresis loops and thermal dependence of the magnetization were measured in the range 2–300 K using a Quantum Design Magnetic Property Measurement System (MPMS 3).

XMCD measurements were performed on the I06 beamline at Diamond Light Source to measure either the atomic magnetic moments of Fe and Mn (using total electron yield detection) or element-selective hysteresis loops (using fluorescence detection). Total electron yield essentially probes surface magnetisation (due to the ∼5 nm electron escape length), while fluorescence detection probes to a depth of ∼200 nm and allows accurate measurements in variable magnetic fields.

To determine the magnetic moments, soft X-ray absorption spectra (XAS) near the Fe and Mn L_3,2_ edges were obtained at 1.6 K for parallel (I^+^) and antiparallel (I^−^) alignment of photon helicity with respect to the applied magnetic field. Magnetic fields in the range ±6 T were applied to magnetize the sample, at an angle 75° with respect to the film normal. The XMCD signal (I^+^–I^−^) was then analyzed using XMCD sum rules^51–53^ to extract the overall magnetic moments of Fe and Mn and separate their orbital and spin contributions for two samples of Fe_1.75_Mn_1.25_Al grown in the same deposition run. A correction factor of 1.47 was used to adjust the Mn spin moment in order to remove intermixing complications between the L_3_ and L_2_ edges.^54^

## Results

The XRD patterns of the as-deposited Fe_2−*x*_Mn_1+*x*_Al films (ESI Note 1†) show only principal reflections of the type (*h* + *k* + *l*)/2 = 2*n*, consistent with the films being isotropic nanocrystals that display the disordered A2 Heusler structure and no traces of spurious phases.^55^ After heat-treatment, the (111) and (200) superlattice reflections appear with intensities that vary strongly with *x*, consistent with the creation of chemical order due to the formation of the B2 and L2_1_ phases (Fig. 1a).

For the varying Mn content of the heat treated films, we have performed a quantitative analysis of the XRD patterns to determine the degree of B2 and L2_1_ ordering *via* the order parameters *S*_B2_ and *S*_L2_1__.^56^ To minimize experimental uncertainties, we prepared and analyzed three equivalent samples for each value of *x*. For each sample we measured the peak intensities *I*_111_(*I*_200_) relative to *I*_220_, *I*_400_ and *I*_422_, thus obtaining 3 × 3 = 9 independent measurements of *S*_L2_1__ (*S*_B2_)^56^ for each value of *x*. We find that the average of *S*_L2_1__ for these nine measurements decreases with *x*, while the average of *S*_B2_ increases, showing that Mn enrichment favors the L2_1_ → B2 transformation (Fig. 1b). Therefore, with the increasing Mn content, Fe is more likely to occupy the Fe sites (8c) implying that *X*–*YZ* disorder decreases, while Mn and Al are more likely to exchange their sites (4a and 4b respectively) implying that *Y*–*Z* disorder increases.

**Fig. 2:**
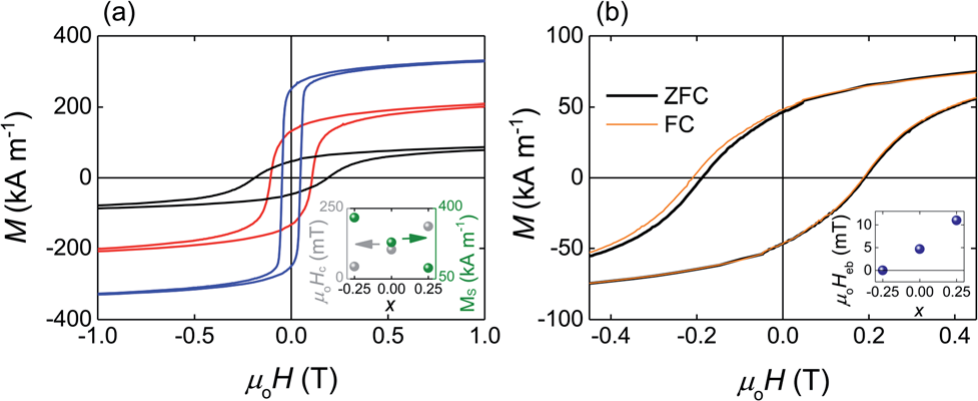
Saturation magnetization, coercivity and exchange bias as a function of composition. (a) Hysteresis loops measured at *T* = 4 K after cooling in zero magnetic field for Fe_2. 25_Mn_0.75_Al (blue), Fe_2_MnAl (red) and Fe_1.75_Mn_1.25_Al (black). Inset: coercive field *μ*_0_*H*_c_ (grey) and saturation magnetization *M*_s_ (green) as a function of composition *x* at *T* = 4 K. (b) Hysteresis loops for the Fe_1.75_Mn_1.25_Al film measured at *T* = 4 K after cooling in a zero (thick black line) and in a 600 mT (thin orange line) applied magnetic field. Inset: exchange-bias field *μ*_0_*H*_eb_ measured at *T* = 4 K as a function of composition. All the presented hysteresis loops are zooms of experimental loops obtained by sweeping the applied magnetic field in the range ±7 T.

The SQUID hysteresis loops measured at *T* = 4 K for the heat-treated Fe_2−*x*_Mn_1+*x*_Al (*x* = −0.25, 0, 0.25) films (Fig. 2a) show that the coercivity increases linearly with the increasing Mn content (from 47 mT for Fe_2.25_Mn_0.75_Al to 186 mT for Fe_1.75_Mn_1.25_Al) while the saturation magnetization decreases linearly (from 360 kA m^−1^ for Fe_2.25_Mn_0.75_Al to 110 kA m^−1^ for Fe_1.75_Mn_1.25_Al) as previously observed in bulk samples of a similar composition.^43^

After field-cooling to *T* = 4 K, the Fe_1.75_Mn_1.25_Al film shows the exchange-bias effect (Fig. 2b) with the exchange-bias field *H*_eb_ = (*H*_c1_ − *H*_c2_)/2 = 11.0 ± 0.2 mT (*H*_eb_ = 36.0 ± 0.5 mT after field cooling to *T* = 2 K, ESI Note 2†). The shift of the hysteresis loop is apparent in the second quadrant because of the circumstantial increase in coercivity. After cooling to *T* = 4 K, *H*_eb_ = 4.7 ± 0.7 mT for the Fe_2_MnAl film and *H*_eb_ = 0.0 ± 0.2 mT for the Fe_2.25_Mn_0.75_Al film, showing that *H*_eb_ decreases linearly with the decreasing Mn content (inset Fig. 2b).

**Fig. 3:**
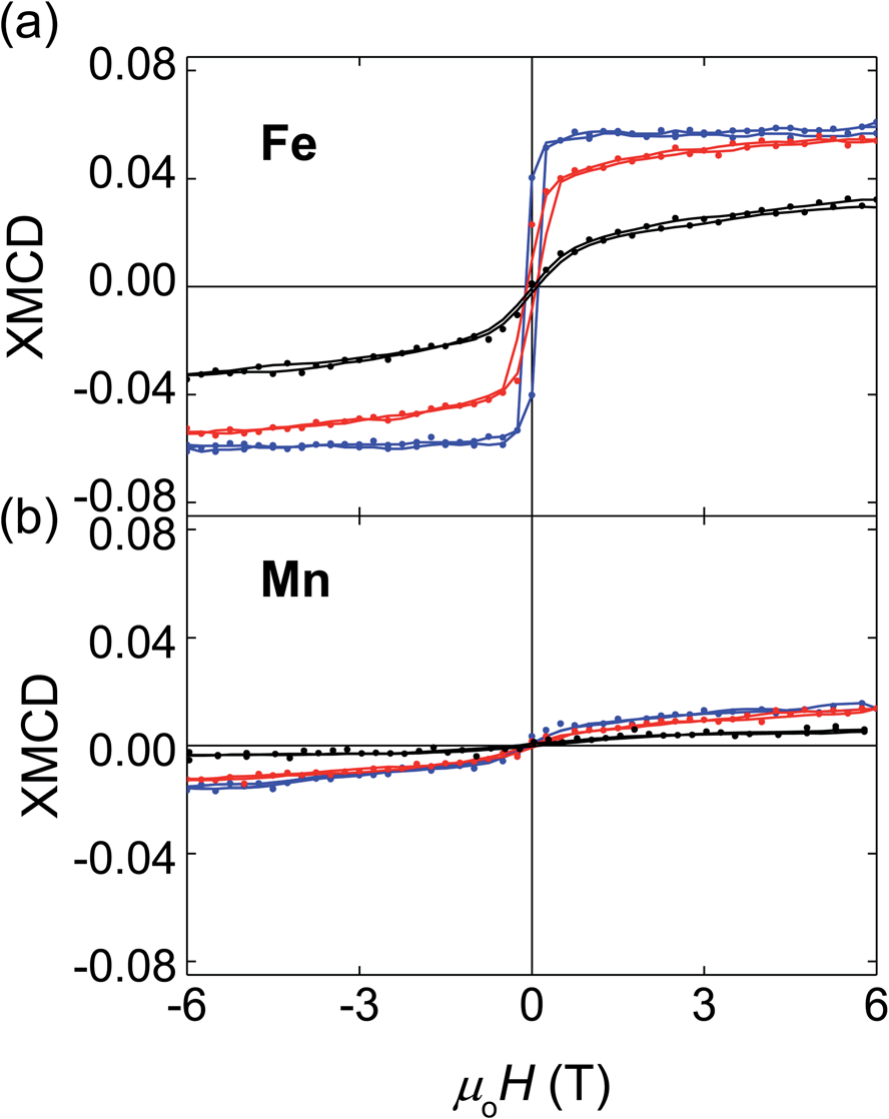
XMCD element selective hysteresis loops as a function of composition. (a) Fe and (b) Mn XMCD hysteresis loops measured at *T* = 1.6 K for Fe_1.75_Mn_1.25_Al (black), Fe_2_MnAl (red) and Fe_2.25_Mn_0.75_Al (blue). Points and solid lines represent raw and smoothed data respectively.

We have used XMCD to investigate the element-specific magnetic behaviour of these films. For films of all compositions, the saturated XMCD signal of the element selective hysteresis loops is ∼5 times larger for Fe (Fig. 3a) than for Mn (Fig. 3b), showing that Fe provides the dominant contribution to the overall magnetic moment. Moreover, with the increasing Mn content, both Fe and Mn moments decrease, in agreement with the decrease of the overall magnetic moment detected by SQUID magnetometry. With the increasing Mn content, the Fe XMCD loops show a dramatic decrease in remanent magnetization and low-field susceptibility, accompanied by a progressive increase in high-field susceptibility. These changes are consistent with the hypothesis that part of the XMCD signal increasingly originates from a canted AFM phase. Similarly clear trends with the increasing Mn content are not evident in the Mn XMCD loops because of the poorer signal to noise ratio.

**Fig. 4:**
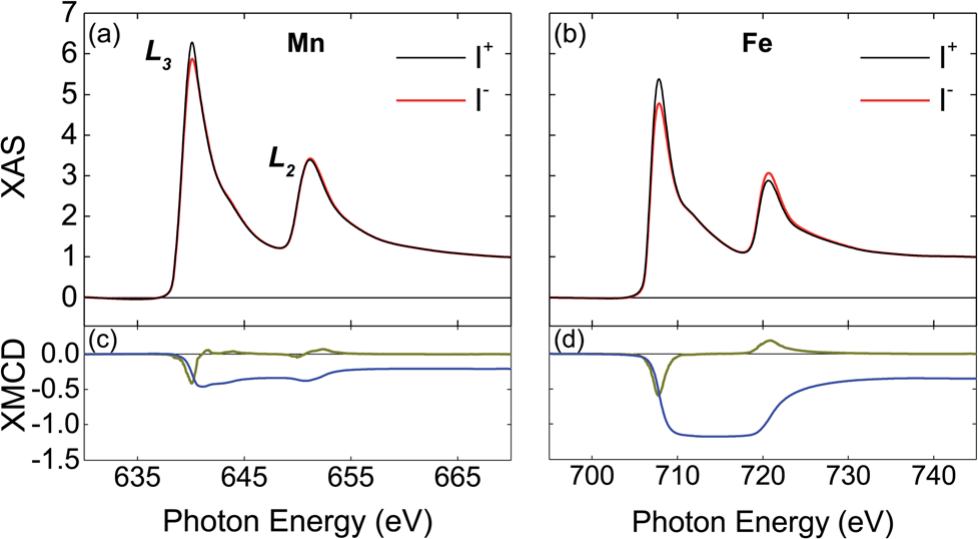
XAS and XMCD spectra for Fe_1.75_Mn_1.25_Al. For the (a and c) Mn and (b and d) Fe L_2_ and L_3_ absorption edges, we present (a and b) X-ray absorption spectra measured with right (black) and left (red) circularly polarized light, (c and d) XMCD spectrum (green) and the integral of the XMCD spectrum (blue). All data measured at *T* = 1.6 K in a 6 T applied magnetic field.

For the Fe_1.75_Mn_1.25_Al film, analysis of the XMCD data (Fig. 4c and d) using sum rules^51–53^ yields 0.60 ± 0.03 *μ*_B_ for the magnetic moment of Fe, 0.15 ± 0.06 *μ*_B_ for Mn, and 1.25 ± 0.08 *μ*_B_/f.u. for the total magnetic moment (we have used 3.4 and 4.5 for the 3d-hole number for Mn and Fe respectively^57,58^). Table 1 summarizes the *m*_spin_, *m*_orb_ and *m*_tot_ for the Fe_1.75_Mn_1.25_Al film. *m*_spin_ dominates the total magnetic moment due to orbital hybridization and the orbital moment has been shown to be negligible.^59^ The trend is also consistent with previous XMCD reports for Fe_2_MnAl.^60^

**Table 1:** Summary of the XMCD sum rule analysis for the Fe_1.75_Mn_1.25_Al film (*m*_spin_, *m*_orb_ and *m*_tot_ (*μ*_B_) denote the measured spin, orbital and total magnetic moments respectively)

	Fe	Mn
*m*_spin_	0.57 ± 0.01	0.12 ± 0.01
*m*_orb_	0.03 ± 0.03	0.03 ± 0.06
*m*_tot_	0.60 ± 0.03	0.15 ± 0.06

Given that both the Fe and the Mn moments are positive, we conclude that ferromagnetic Fe–Mn interactions are predominant. We could not apply XMCD sum rules to the films richer in Fe because their XAS spectra measured in total electron yield did not possess the required high-quality, due to some degree of surface oxidation (ESI Note 3†). Our Fe_1.75_Mn_1.25_Al film did not show any trace of surface oxidation (even though it is the richest in Mn, which has higher oxygen affinity than Fe) likely because it displays low surface roughness, such that protection from the 2.5 nm-thick Al capping layer is very effective (ESI Note 4†).

For the increasing Mn content, changes in the magnetic properties of our Heusler films can be explained by the formation of an antiferromagnetic phase. This antiferromagnetic phase can only be the B2 phase, given that our XRD analysis shows that a partial L2_1_ → B2 phase transformation is the only change to the crystal structure that is induced by the increasing Mn content. The L2_1_ phase of bulk Fe_2_MnAl is ferromagnetic^43^ while the B2 phase can be argued to be antiferromagnetic.^61,62^ Therefore, the properties of our films are understood in terms of a mixed state, where ferromagnetism from the L2_1_ phase coexists with antiferromagnetism from the B2 phase as in bulk Ni_2_MnAl.^61,62^

For bulk Heusler alloys in the L2_1_–B2 mixed state, the magnetization curves obtained as a function of temperature after cooling with (FC) and without (ZFC) an applied magnetic field are split.^43,62^ This splitting is incompatible with a collinear homogeneous antiferromagnetic state, and has been attributed to pinning of ferromagnetic moments *via* antiferromagnetic anisotropy.^43,62,63^ This suggestion implies that the L2_1_–B2 mixed state is heterogeneous, with separated ferromagnetic and antiferromagnetic regions. However, in the absence of direct observations, the microstructure of the mixed L2_1_–B2 Heusler state has remained so far elusive.

**Fig. 5:**
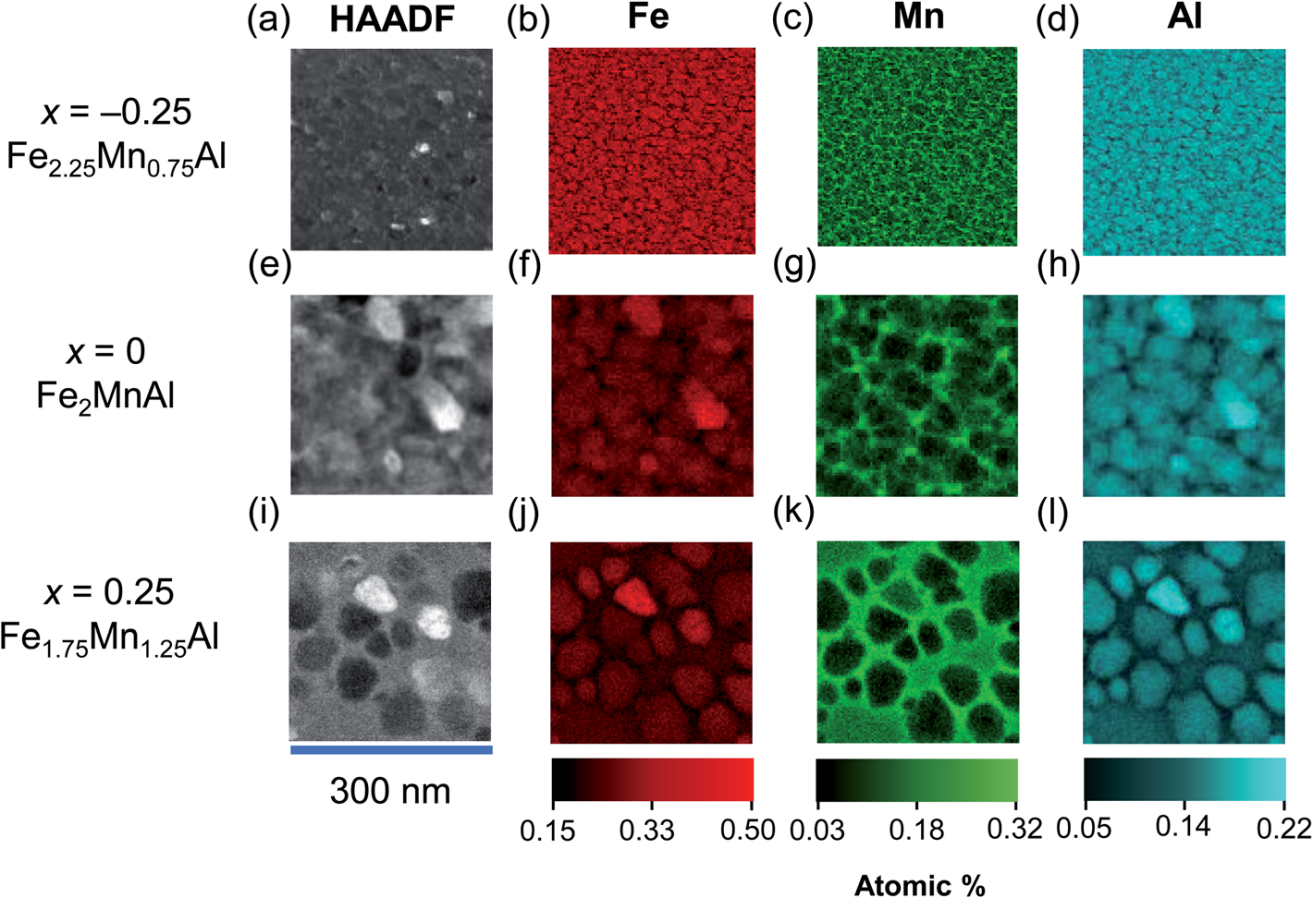
Nanosegregation in Fe_2−*x*_Mn_1+*x*_Al films with exchange-bias. HAADF images (a, e and i) for (a–d) Fe_2.25_Mn_0.75_Al (e–h) Fe_2_MnAl and (i–l) Fe_1.75_Mn_1.25_Al and elemental quantitative EDX maps for (b, f and j) Fe, (c, g and k) Mn and (d, h and l) Al.

In our nanocrystalline films, we observe split ZFC–FC curves (ESI Note 5†), consistent with the L2_1_–B2 mixed state and an heterogeneous microstructure. To check this conclusion directly, we have performed a STEM study with composition analysis (Fig. 5). The STEM-HAADF images for the increasing Mn content (Fig. 5a–c) show that the films are nanocrystalline with an average grain size that increases from 28 ± 2 nm (for *x* = −0.25) to 82 ± 3 nm (for *x* = 0.25). The corresponding elemental maps show that with the increasing Mn content the grains become progressively richer in Fe (Fig. 5d–f) and poorer in Mn (Fig. 5g–i), while the intergrain volume becomes richer in Mn and poorer in Fe. The elemental maps for Al (Fig. 5j–l) indicate a behavior similar to that of Fe. The elemental maps suggest that substituting Mn for Fe drives the nanosegregation of a Mn-rich phase outside the grains, with an Fe-rich phase inside the grains.

**Fig. 6:**
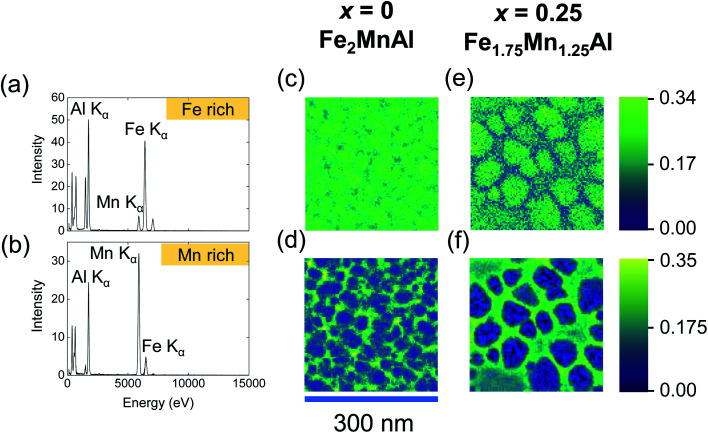
Compositions of the segregated phases in Fe_2_MnAl and Fe_1.75_Mn_1.25_Al films by NMF analysis of the EDX spectra. EDX spectra for the (a) Fe-rich and (b) Mn-rich NMF factors and the corresponding loadings (spatial maps) are shown for (c and d) Fe_2_MnAl and (e and f) Fe_1.75_Mn_1.25_Al films. The colour scale intensity indicates the weight of the factor for a given pixel.

For the Fe_2_MnAl and Fe_1.75_Mn_1.25_Al films, that show the exchange-bias effect, NMF analysis (Fig. 6a and b) yields two separate Fe–Mn–Al ternary phases, one Fe-rich and a Mn-rich. The compositions of these ternary phases (Table 2) should be taken as qualitative, because they can be affected by local inhomogeneities in EDX artifacts, such as fluorescence peaks and different local absorptions of emitted X-rays. While the quantification of larger-area integrated EDX data provides a more accurate estimate of the film composition, NMF demonstrates the segregation into the two phases and unambiguously shows that they are ternary compounds of Fe, Mn and Al. For both the Fe_2_MnAl and the Fe_1.75_Mn_1.25_Al films, the spatial maps (loadings) of the Fe-rich and Mn-rich phases show that intragrain volumes are Fe-rich, while intergrain volumes are Mn-rich. For the Fe_2.25_Mn_0.75_Al film (data not shown), the Fe-rich factor did not show clear grains as they were homogeneously distributed over length scales consistent with negligible nanosegregation for this sample.

**Table 2:** Compositions of the nanosegregated Fe-rich and Mn-rich phases determined using the NMF algorithm for the Fe_2_MnAl and Fe_1.75_Mn_1.25_Al films that present exchange-bias

Film	Fe-rich phase	Mn-rich phase
Fe_2_MnAl	Fe_2.3±0.1_Mn_0.56±0.05_Al_1.10±0.05_	Fe_0.44±0.02_Mn_3.3±0.2_Al_0.23±0.01_
Fe_1.75_Mn_1.25_Al	Fe_2.25±0.1_Mn_0.85±0.05_Al_0.91±0.05_	Fe_0.45±0.02_Mn_3.4±0.2_Al_0.15±0.01_

We now combine the microstructural information that we obtained by STEM with quantitative SQUID and XMCD magnetometry, to show that the exchange-bias effect originates from the observed nanosegregation process.

For the Fe_1.75_Mn_1.25_Al film, the total magnetic moment deduced from the SQUID data (using the volume of the whole film) is 0.55 ± 0.01 *μ*_B_/f.u., *i.e.* ∼44% of the value 1.25 ± 0.08 *μ*_B_/f.u. obtained by XMCD. This discrepancy can be solved by noting that, due to phase segregation, the Fe-rich grains constitute ∼45% of the field of view of a 2 μm × 2 μm STEM image (zoomed image shown in Fig. 5i). Using the reduced volume occupied by the Fe-rich phase instead of the whole film, SQUID magnetometry yields a total magnetic moment of 1.22 ± 0.06 *μ*_B_/f.u. in good agreement with XMCD. Therefore, only the Fe-rich phase is ferromagnetic, while the Mn-rich phase is antiferromagnetic.

The Fe-rich grains constitute ∼71% of the field of view of a 2 μm × 2 μm STEM image of the Fe_2_MnAl film (zoomed image shown in Fig. 5f). Therefore the packing fraction of the Mn-rich (Fe-rich) phase increases (decreases) with the Mn content from ∼29% (71%) in the Fe_2_MnAl film, to ∼55% (45%) in the Fe_1.75_Mn_1.25_Al film. This trend mirrors the increase (decrease) of the *S*_B2_ (*S*_L2_1__) order parameter with the increasing Mn content (Fig. 1b) which suggests that the Mn-rich segregated phase is the B2 phase, while the Fe-rich segregated phase is the L2_1_ phase. Consistently, XRD shows no sign of other phases, either crystalline (*e.g.* β-Mn phase^64^) or amorphous (as expected, in view of our magnetic data and heat treatments).

Therefore, our samples present an heterogeneous L2_1_–B2 mixed state, with ferromagnetic nanograins surrounded by antiferromagnetic regions. This microstructure is similar to core–shell systems where the exchange-bias results from interfacial exchange-coupling between the ferromagnetic core nanoparticles and the surrounding antiferromagnetic matrix.^15^

In summary, we have presented the first study of the magnetic and structural properties of Heusler Fe_2_MnAl individual thin films. The films of interest display a mixed L2_1_–B2 crystallographic structure (induced by post-deposition heat treatments) and are phase-separated due to a nanosegregation process which depends on the Mn concentration. This nanosegregation creates a microstructure similar to core/shell systems, and thus establishes an exchange-bias effect that can be tuned by varying the Mn concentration. We show that exchange-bias arises from interfacial exchange-coupling between the Fe-rich ferromagnetic L2_1_ phase and the Mn-rich antiferromagnetic B2 phase. Our report of exchange-bias in an environmentally friendly nanocomposite film should inspire the development of single-layer spin valve devices which are sustainable.

## Conflicts of interest

There are no conflicts to declare.

## Supplementary Material

NA-002-C9NA00689C-s001
