# Supplementary material for: Exchange-bias via nanosegregation in novel Fe2−xMn1+xAl (x = −0.25, 0, 0.25) Heusler films
Source: Nanoscale Adv. 2020 May 1;2(6):2602–9. doi: 10.1039/c9na00689c (PMC9417214; doi:10.1039/c9na00689c)
Supplement: NA-002-C9NA00689C-s001 [file NA-002-C9NA00689C-s001.pdf]

Electronic Supplementary Material (ESI) for Nanoscale Advances.
This journal is © The Royal Society of Chemistry 2020

# Supplementary information

# for

# Exchange-bias via nanosegregation in novel $Fe_{2-x}Mn_{1+x}Al$ ($x$ = –0.25, 0, 0.25) Heusler films

S. Kurdi[1,*], M. Ghidini[2,3,1,‡], G. Divitini[1], B. Nair[1], A. Kursumovic[1], P. Tiberto[4], S.S. Dhesi[3], Z.H. Barber[1]

[1] Department of Materials Science and Metallurgy, University of Cambridge, CB3 0FS Cambridge, United Kingdom.

[2] Department of Physics, Mathematics and Computer Science, University of Parma, 43130 Parma, Italy.

[3] Diamond Light Source, Chilton, Didcot, OX11 0DE Oxfordshire, United Kingdom

[4] The National Institute for Metrological Research (INRIM), 10135 Torino, Italy.

* sk862@cantab.ac.uk

‡ massimo.ghidini@unipr.it

## Contents

**Note1 As-deposited Film Characterization**

**Note 2 Exchange-Bias at $T$ = 2 K for the $Fe_{1.75}Mn_{1.25}Al$ film**

**Note 3 Mn and Fe XAS**

**Note 4 Morphology and Surface Roughness**

**Note 5 Curie temperatures from ZFC/FC curves**

## Supplementary Note 1

### As-deposited Film Characterization

**XRD of the as-deposited $Fe_{2-x}Mn_{1+x}Al$ films.** Fig. S1 shows only principal reflections (of the type (h+k+l)/2 = 2n). The absence of superlattice reflections shows that the films possess the disordered *A*2 Heusler structure. Peaks are broad, which suggests that the as-deposited films have a very small crystallite size, as confirmed by STEM (Fig. S2), which shows homogenous microstructure with grain size ~10 nm or smaller.

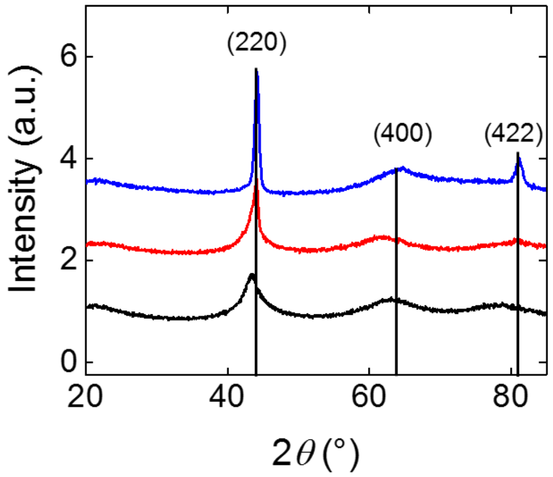


**Figure S1. XRD of the as deposited $Fe_{2-x}Mn_{1+x}Al$ films ($x$ = –0.25, 0, 0.25).** XRD patterns for compositions $Fe_{2.25}Mn_{0.75}Al$ (blue), $Fe_2MnAl$ (red) and $Fe_{1.75}Mn_{1.25}Al$ (black). The vertical black lines represent the expected positions of the fundamental peaks.

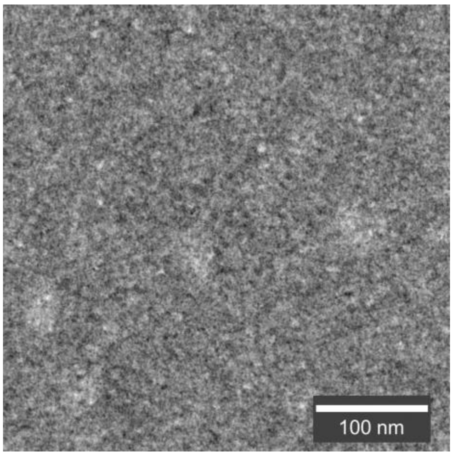


**Figure S2.** High-angle annular dark-field STEM image of the as deposited $Fe_2MnAl$ film.

## Supplementary Note 2

## Exchange-Bias at $T$ = 2 K for the $Fe_{1.75}Mn_{1.25}Al$ film

For all the samples discussed in this paper, the highest value of 36 ± 0.05 mT for the exchange-bias field, was measured at $T$ = 2 K after field cooling in a 600 mT field (Fig. S3).

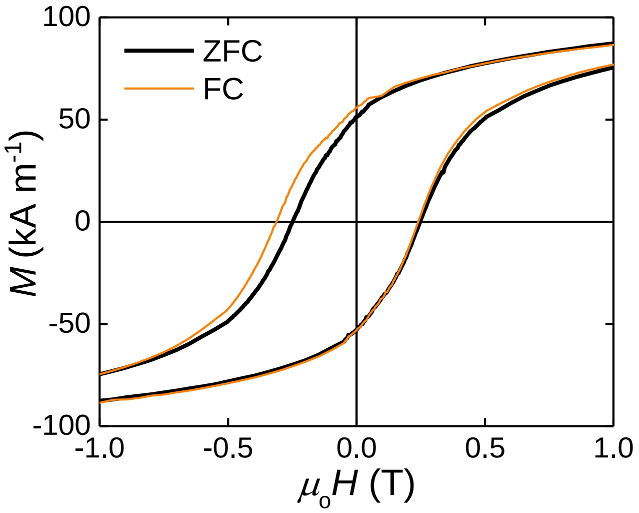


**Figure S3.** Zooms of the hysteresis loops for the $Fe_{1.75}Mn_{1.25}Al$ film measured at $T$ = 2 K after cooling in zero applied magnetic field (thick black line) and in a 600 mT applied magnetic field (thin orange line). Both hysteresis loops were measured sweeping the magnetic field in the range ± 7 T.

## Supplementary Note 3

## Mn and Fe XAS

For all film compositions, here we present typical XAS scans measured in total electron yield at $T$ =1.6 K and in a 6 T applied magnetic field. Multiplet peaks are present in the Mn scans for the $Fe_2MnAl$ and $Fe_{2.25}Mn_{0.75}Al$ (as shown by arrows) indicating the presence of some degree of surface oxidation. For this reason, as explained in the main text, it has not been possible to apply XMCD sum rules to these samples. However, these spurious peaks are absent in the corresponding data for the $Fe_{1.75}Mn_{1.25}Al$ film and the high quality of the XAS spectra has allowed the quantitative XMCD analysis that we presented in the main paper (Fig. 4).

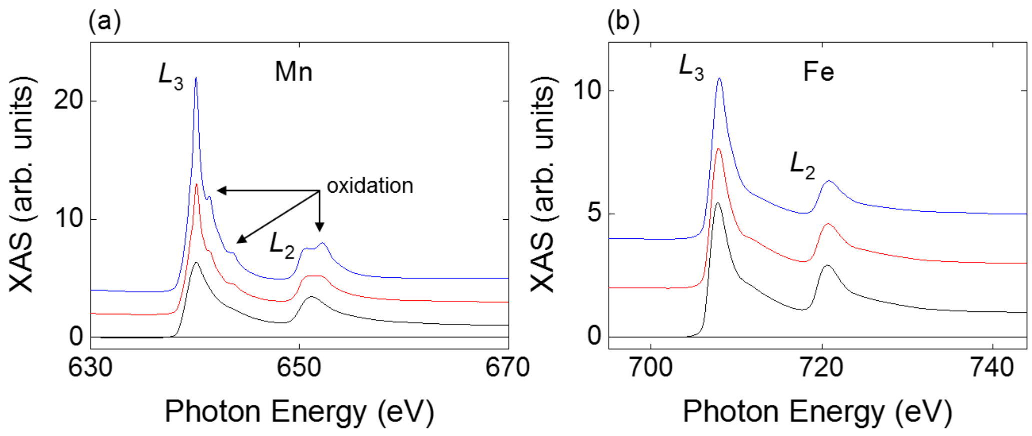


**Figure S4.** (a) Mn and (b) Fe raw XAS scans (with photon helicity parallel to magnetic field, $I^+$) measured at $T$ = 1.6 K in a 6 T field for $Fe_{1.75}Mn_{1.25}Al$ (black), $Fe_2MnAl$ (red) and $Fe_{2.25}Mn_{0.75}Al$ (blue). Black arrows point to spurious oxide peaks in the Mn XAS scans for $Fe_2MnAl$ and $Fe_{2.25}Mn_{0.75}Al$.

## Supplementary Note 4

### Morphology and Surface Roughness

Here we present AFM scans for all film compositions (Fig. S5). Root-mean-square squareness $R_{rms}$ (as determined from the three 1×1 μm$^2$ scans below) is 1.07 ± 0.02 nm, 3.56 ± 0.1 nm and 4.10 ± 0.04 nm for $Fe_{1.75}Mn_{1.25}Al$, $Fe_2MnAl$ and $Fe_{2.25}Mn_{0.75}Al$ respectively. The $Fe_{1.75}Mn_{1.25}Al$ films show the lowest peak-to-peak roughness: 10.5 ± 0.5 nm compared to 25.5 ± 1.0 nm for $Fe_2MnAl$ and 26.5 ± 0.8 nm for $Fe_{2.25}Mn_{0.75}Al$. The increase of Fe (decrease in Mn) content shows a surface microstructure with finer grain size.

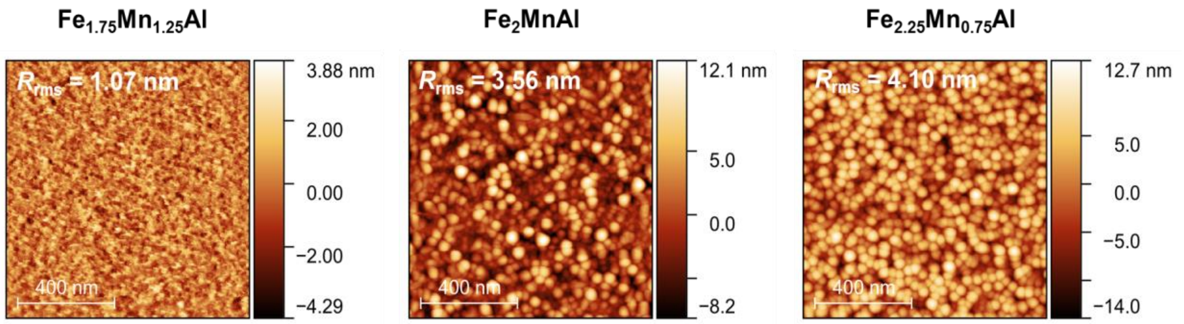


**Figure S5. Surface morphology for the $Fe_{2-x}Mn_{1+x}Al$ films.** Roughness ($R_{rms}$) was determined from three 1 x 1 μm$^2$ scans.

## Supplementary Note 5

### Curie temperatures from ZFC/FC curves

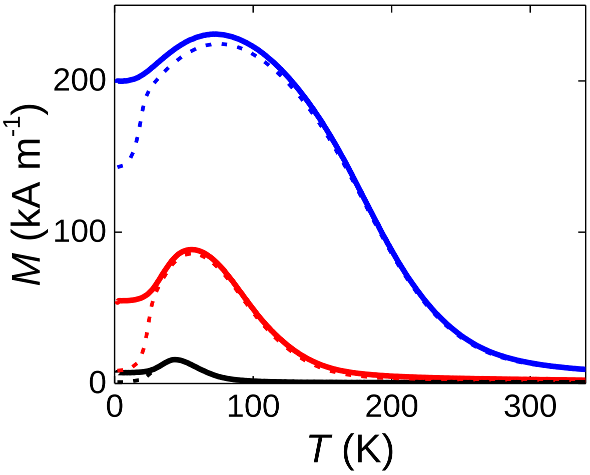


**Figure S6.** Thermal dependence of the magnetization as measured after cooling in zero-field (dashed lines) and while cooling in a field $\mu_0 H$ = 5mT (solid lines), for the films with nominal composition $Fe_{2.25}Mn_{0.75}Al$ (blue), $Fe_2MnAl$ (red) and $Fe_{1.75}Mn_{1.25}Al$ (black).

The $T_c$ values were extracted from Fig. S6 using the two-tangent method[1], and are summarized in Table S1 below for all film compositions.

**Table S1.** The Curie temperature ($T_c$) of the $Fe_{2.25}Mn_{0.75}Al$, $Fe_2MnAl$ and $Fe_{1.75}Mn_{1.25}Al$ films.

| **Film** | $T_c$ **[K]** |
|---|---|
| $Fe_{2.25}Mn_{0.75}Al$ | 268 |
| $Fe_2MnAl$ | 150 |
| $Fe_{1.75}Mn_{1.25}Al$ | 83 |

As in previous studies[2,3] we observe that $T_c$ decreases with decreasing Fe concentration (mirroring as expected the observed decrease in magnetic moment). This behaviour has been attributed to the ferromagnetic nature of the dominant Fe-Fe interactions. For the $Fe_2MnAl$ sample we observe $T_c$ ~ 150 K which is lower than the values reported for bulk samples in some studies[4,5], but comparable to another study[2]. This large spread in the reported $T_c$ values is not surprising in Heusler alloys and is likely related to the role of chemical disorder[6], but more specifically in nanocrystalline films $T_c$ is also affected by extrinsic factors such as strain and size effects.

The suppression of ferromagnetism at room temperature could in principle be due to superparamagnetism, but we can rule this out in view of our STEM data, showing that the sample with the smallest average grain size also displays the highest $T_c$.

**References**


1 K. Fabian, V. P. Shcherbakov and S. A. McEnroe, *Geochem. Geophys.*, 2013, **14**, 947–961.

2 Z. Liu, X. Ma, F. Meng and G. Wu, *J. Alloys Compd.*, 2011, **509**, 3219–3222.

3 H. Bremers, J. Hesse, H. Ahlers, J. Sievert and D. Zachmann, *J. Alloys Compd.*, 2004, **366**, 67–75.

4 C. Paduani, A. Migliavacca, W. E. Pöttker, J. Schaf, J. C. Krause, J. D. Ardisson, C. A. Samudio Pérez, A. Y. Takeuchi and M. I. Yoshida, *Phys. B Condens. Matter*, 2007, **398**, 60–64.

5 K. H. J. Buschow, P. Engen and P. G. van Engen, *J. Magn. Magn. Mater.*, 1981, **25**, 90–96.

6 J. Karel, J. E. Fischer, S. Fabbrici, E. Pippel, P. Werner, M. Vinicius Castergnaro, P. Adler, S. Ouardi, B. Balke, G. H. Fecher, J. Morais, F. Albertini, S. S. P. Parkin and C. Felser, *J. Mater. Chem. C*, 2017, **5**, 4388–4392.
